# SUN-domain proteins of the malaria parasite *Plasmodium falciparum* are essential for proper nuclear division and DNA repair

**DOI:** 10.1128/mbio.00216-25

**Published:** 2025-03-05

**Authors:** Sofiya Kandelis-Shalev, Manish Goyal, Tal Elam, Shany Assaraf, Noa Dahan, Omer Farchi, Eduard Berenshtein, Ron Dzikowski

**Affiliations:** 1 Department of Microbiology and Molecular Genetics, The Kuvin Center for the Study of Infectious and Tropical Diseases, IMRIC, The Hebrew University-Hadassah Medical School, Jerusalem, Israel; 2 Core facility of The Hebrew University-Hadassah Medical School, Jerusalem, Israel; The George Washington University Milken Institute of Public Health, Washington, DC, USA

**Keywords:** malaria, *Plasmodium falciparum*, nuclear envelope, LINC complex, sun-domain protein, DNA damage

## Abstract

The protozoan parasite *Plasmodium falciparum*, which is responsible for the deadliest form of human malaria, accounts for over half a million deaths a year. These parasites proliferate in human red blood cells by consecutive rounds of closed mitoses called schizogony. Their virulence is attributed to their ability to modify the infected red cells to adhere to the vascular endothelium and to evade immunity through antigenic switches. Spatial dynamics at the nuclear periphery were associated with the regulation of processes that enable the parasites to establish long-term infection. However, our knowledge of components of the nuclear envelope (NE) in *Plasmodium* remains limited. One of the major protein complexes at the NE is the linker of nucleoskeleton and cytoskeleton (LINC) complex that forms a connecting bridge between the cytoplasm and the nucleus through the interaction of SUN and KASH domain proteins. Here, we have identified two SUN-domain proteins as possible components of the LINC complex of *P. falciparum* and show that their proper expression is essential for the parasite’s proliferation in human red blood cells, and their depletion leads to the formation of membranous whorls and morphological changes of the NE. In addition, their differential expression highlights different functions at the nuclear periphery as PfSUN2 is specifically associated with heterochromatin, while PfSUN1 expression is essential for activation of the DNA damage response. Our data provide indications for the involvement of the LINC complex in crucial biological processes in the intraerythrocytic development cycle of malaria parasites.

**IMPORTANCE:**

*Plasmodium falciparum*, the parasite causing the deadliest form of malaria, is able to thrive in its human host by tight regulation of cellular processes, orchestrating nuclear dynamics with cytoplasmic machineries that are separated by the nuclear envelope. One of the major protein complexes that connect nuclear and cytoplasmic processes in eukaryotes is the linker of nucleoskeleton and cytoskeleton (LINC) complex. However, while the nuclear periphery of *P. falciparum* was implicated in several important functions, the role of the LINC complex in Plasmodium biology is unknown. Here, we identify two components of *P. falciparum* LINC complex and demonstrate that they are essential for the parasites’ proliferation in human blood, and their depletion leads to the formation of morphological changes in the cell. In addition, the two components have different functions in activating the DNA damage response and in their association with heterochromatin. Our data provide evidence for their essential roles in the parasites’ cell cycle.

## INTRODUCTION

Malaria caused by protozoan parasites of the genus *Plasmodium* remains one of the leading causes of morbidity and mortality worldwide. These parasites are estimated to infect over 200 million people each year, resulting in over 600,000 documented deaths (1). *Plasmodium falciparum* is responsible for the deadliest form of human malaria that accounts for over 90% of malaria deaths, primarily of young children and pregnant women. The lack of an effective vaccine as well as the ability of the parasite to develop resistance to effectively all anti-malarial drugs keeps malaria as a major health and economic burden in many endemic countries.

*P. falciparum* has a complex life cycle, during which it undergoes unique replication cycles and morphological changes to adapt to different hosts and changing environments. These changes are mediated by tight regulation of cell cycle-dependent gene expression patterns (2) (3). Like any other eukaryote, the parasite’s ability to proliferate and thrive in its human host depends on the precise orchestration of biological processes and molecular exchange between the cell’s nucleus and the cytoplasm, which are separated by the nuclear envelope (NE). Evidently, the NE is not only a passive nuclear boundary but is also involved in controlling nuclear and cellular processes and plays critical roles in transferring signals from the cytoplasm and the extracellular environment into the nucleus (4–7).

One of the major protein complexes that connect nuclear and cytoplasmic processes is the linker of nucleoskeleton and cytoskeleton (LINC) protein complex (8–11). The LINC complex spans the NE across the fluid lumen and forms a direct mechanical connection between the nucleus and the cytoplasm. LINC complexes are composed of SUN-domain proteins, residents of the inner nuclear membrane (INM), that interact with KASH domain proteins (Nesprins) that are embedded in the outer nuclear membrane (ONM). In higher eukaryotes, the LINC complex was demonstrated to form a physical contact between components of the cytoskeleton and the nuclear lamina and was implicated in determining nuclear positioning, migration, and orientation as well as influencing cell division (12). In addition, the LINC complex was implicated in DNA repair (13–15) and in chromosome organization by forming a mechanical link between the cytoskeleton and DNA (16–19). Furthermore, in addition to providing structural integrity for the nucleus, the LINC complex allows for the transfer of mechanical force into the nucleus resulting in mechanotransduction of signals from the cell’s extracellular environment to the nucleus (8, 20).

Very little is known about the components of the NE in *P. falciparum*. These parasites lack conventional lamins, and thus, factors that constitute the nuclear scaffold under the INM remain unknown. In addition, only a few nucleoporins have been characterized, despite the functional conservation of the nuclear pore complex (NPC) throughout eukaryotic evolution. Similar to other components of the NE, little is known about the constituents of the LINC complex in *Plasmodium* or any other apicomplexan parasites. Recent studies have revealed the presence of an alternative LINC-like complex in *Plasmodium berghei*, comprising SUN1 and the newly identified protein ALLC1, which serves a functionally analogous role to KASH-domain proteins (21, 22). The SUN1-ALLC1 complex has been implicated in the organization of the microtubule organizing center (MTOC) and the coordination of mitotic spindle formation, highlighting its critical role in male gametogenesis and malaria transmission.

Here, we identified and characterized two *P. falciparum* SUN-domain proteins, which we called PfSUN1 and PfSUN2. We found that these NE proteins play a role in parasite proliferation during the intraerythrocytic development cycle (IDC). The depletion of expression of both PfSUN1 and PfSUN2 results in increased cellular deformability during schizogony and the appearance of membranous whorls that bud from the NE at earlier stages. Interestingly, we found that PfSUN2 appears to be strongly associated with heterochromatin at the nuclear periphery. Finally, our data indicate that PfSUN1 depletion interferes with the parasite’s ability to activate the DNA damage response (DDR). Our data provide the first functional roles of possible components of the LINC complex in *P. falciparum* biology.

## RESULTS

### Putative SUN-domain proteins in *P. falciparum*

We performed a BLAST search in the *Plasmodium* database (https://plasmodb.org/plasmo/app) (23) using the amino acid sequences of the human orthologs and identified two putative proteins that contain SUN domains, which we named as PfSUN1 (PF3D7_1215100) and PfSUN2 (PF3D7_1439300; Fig. S1). We found that the SUN domain of PfSUN1 displayed higher similarity and structural conservation with that of the human HsSUN2 and 5, while the SUN domain of PfSUN2 shares higher similarity with that of *Arabidopsis thaliana* (Fig. S1A, B, D and E). Both proteins are conserved among *Plasmodium* species (Fig. S1C and F). Multiple sequence alignments of PfSUN1 and PfSUN2 with different SUN domains from evolutionarily distinct organisms indicated that the SUN domains could be separated into two main clades (Fig. S1G left). Interestingly, while PfSUN1 belongs to the same clade as the canonical C’ SUN domain family, PfSUN2 appears to resemble mid-SUN-domain proteins characterized primarily in plants (Fig. S1G right). In both cases, *P. falciparum* SUN-domain proteins contain the structural features of their respective protein clade, i.e., transmembrane (TM) domains, coiled-coil domains (CC), and the SUN domain. This conservation suggests that Plasmodial putative SUN-domain proteins may function as components of the LINC complex, similar to SUN-domain proteins in other organisms.

### PfSUN1 is a nuclear envelope protein

**Fig 1:**
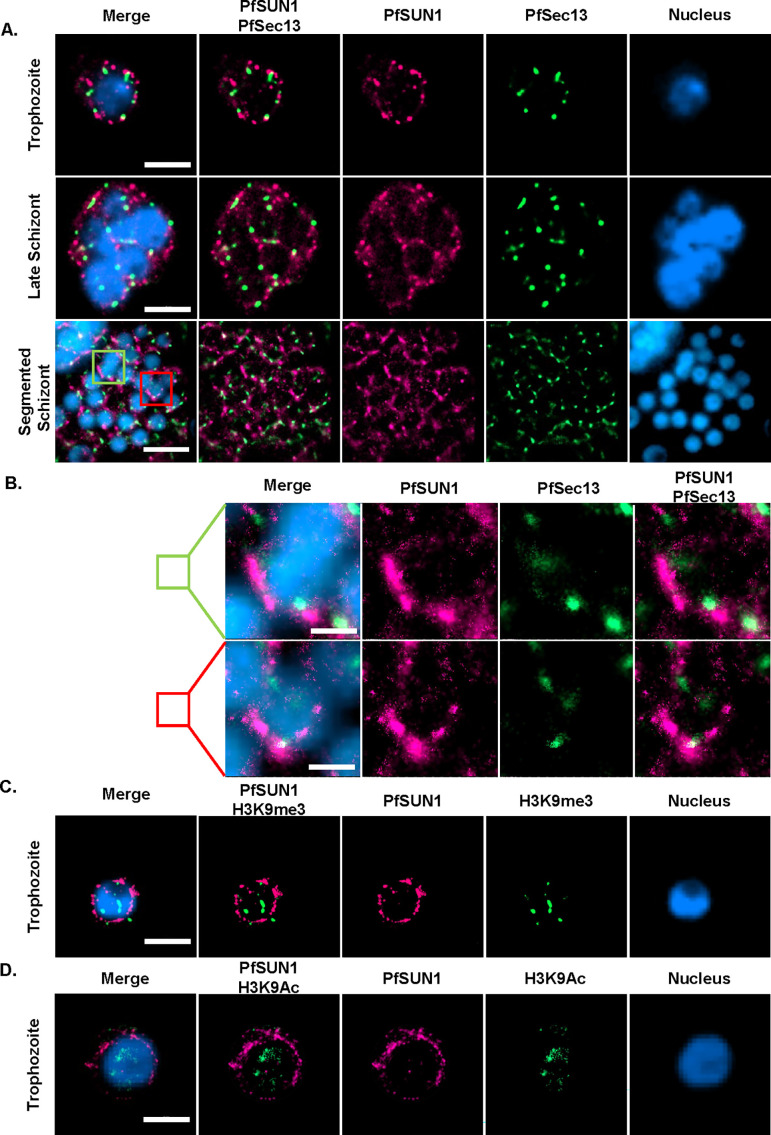
Ectopic PfSUN1 is localized to the nuclear periphery and displays polar accumulation during schizogony. (A) Dual color dSTORM imaging analysis of PfSUN1-GFP (magenta), with PfSEC13-Halo (green) during *P. falciparum* IDC showing their distribution at the nuclear periphery during IDC. Nuclei were stained with YOYO1 dye (blue). Scale bar: 2 µm. (B) High magnification of merozoites from the segmented schizont labeled by green or magenta rectangle shows the polar distribution of the NPC and PfSUN1-GFP. Scale bar: 0.5 µm. (C) dSTORM imaging of PfSUN1 association with heterochromatin and euchromatin histone markers. The upper panel shows PfSUN1-GFP (magenta) localization with the heterochromatin mark H3K9me3 (yellow), and the lower panel shows localization with the euchromatin mark H3K9Ac (green). Scale bar: 2 µm.

Analysis of the available transcriptomic data in https://plasmodb.org/plasmo/app indicated that both SUN-domain proteins are transcribed throughout the parasite’s life cycle with some stage-specific variations in their transcript levels (Fig. S2). To begin characterizing *P. falciparum* putative SUN-domain proteins, we initially expressed PfSUN1-GFP ectopically (Fig. S3A and B) and followed its cellular localization during the parasite’s IDC. We found that PfSUN1-GFP surrounds the DNA staining at the nuclear periphery throughout the IDC (Fig. S3B), suggesting possible localization with the NE. Interestingly, in some nuclei, PfSUN1-GFP is concentrated at distinct foci at the nuclear periphery. To validate its association with the NE, we performed immune-EM and found that PfSUN1-GFP is indeed located at the NE (Fig. S1C). We performed direct stochastic optical reconstruction microscopy (dSTORM) imaging to test its association with the NPC using the nucleoporin PfSec13 as a marker (24). To this end, parasites were transfected with two different episomes expressing either PfSUN1 fused with GFP (PfSUN1-GFP) and PfSEC13 fused with HALO-tag (PfSec13-Halo). We found that both PfSUN1 and PfSec13 surround the nuclear periphery during the IDC (Fig. 1A). Interestingly, while PfSec13 localized to the distinct foci of the NPCs as previously demonstrated, PfSUN1 appears to be surrounding the nucleus in a more continuous pattern, which also contains foci of stronger signal that are often adjacent to the NPC. We have previously observed changes in the directionality of the NPC as mitosis progresses that provided evidence for cytoplasmic influence on NPC localization during the transition from a multinucleated syncytium to multiple autonomous cells (25). Interestingly, we observed a pronounced accumulation of PfSUN1 in late-stage schizonts at the same nuclear pole with the NPC clusters (Fig. 1B). The nuclear periphery of *P. falciparum* includes regions of condensed heterochromatin marked by the histone modification H3K9me3. This histone modification appears to be an epigenetic marker specifically devoted to gene families that undergo clonally variant transcription that localizes to the nuclear periphery (3, 26). Further dSTORM imaging indicated that while it surrounds the nucleus, PfSUN1-GFP does not co-localize with either the heterochromatic marker H3K9me3 or the euchromatin marker H3K9ac (Fig. 1C and D).

**Fig 2:**
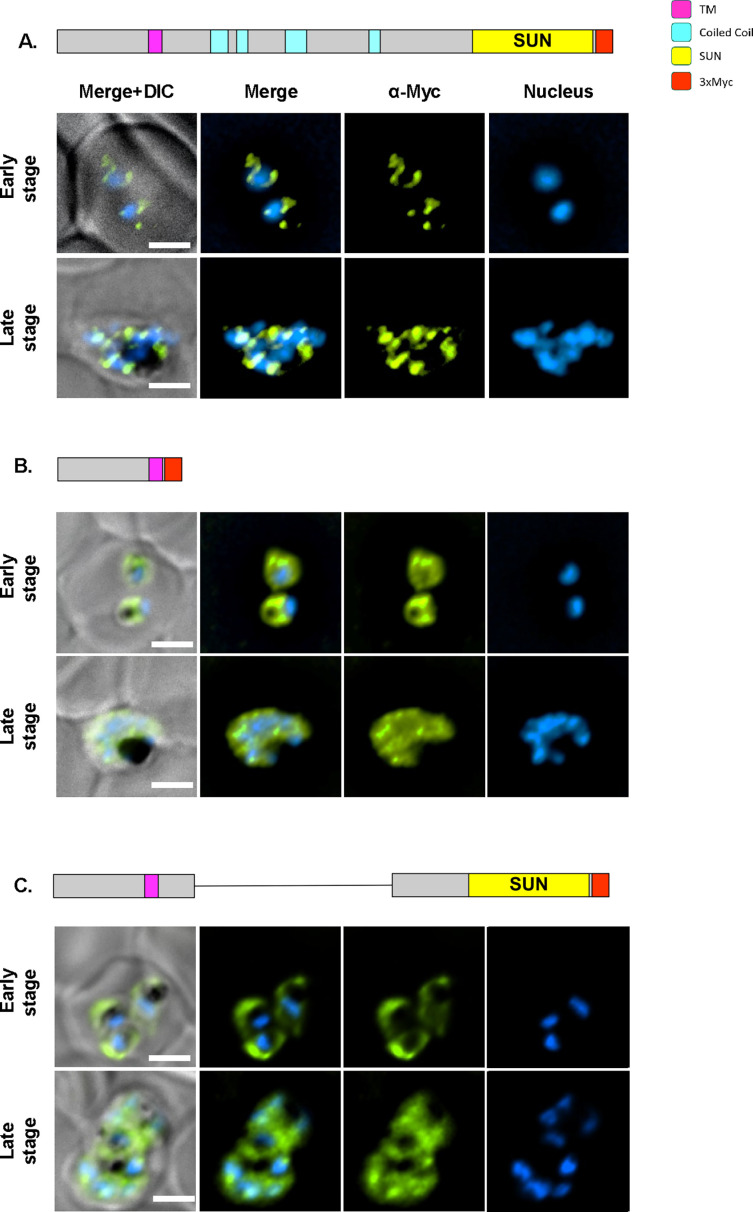
The deletion of PfSUN1 coiled coil motifs results in the loss of NE localization. Immunofluorescence imaging of parasites that ectopically express different deletion mutants of PfSUN1-myc in NF54 parasites. (**A**) Full-length PfSUN1-myc. (**B**) PfSUN1 1–190-myc (expressing the N terminal 190 aa of PfSUN1). (**C**) PfSUN1Δ250–600-myc (coiled-coil domains deleted). Anti-myc antibody labels PfSUN1 mutants (green), nuclei were stained with 4′,6-diamino-2-phenylindole (DAPI; blue). Predicted domain architecture: SUN domain (yellow), TM domain (magenta), coiled-coil domains (cyan), and myc tag (red). Scale bars: 2 µm. DIC, differential interference contrast.

To determine which of the PfSUN1 domains is essential for its nuclear positioning, we performed deletion analysis of the coiled-coil and the SUN domains and imaged parasites expressing different truncated versions of PfSUN1 fused with a *myc* epitope tag (Fig. 2). We found that deletions of the coiled-coil domain as well as deletion of both the coiled-coil and the SUN domain abolished the positioning of PfSUN1 at the nuclear envelope, even though the transmembrane domain was not deleted. These data suggest that the coiled-coil domain is essential for proper positioning of PfSUN1 at the NE.

**Fig 3:**
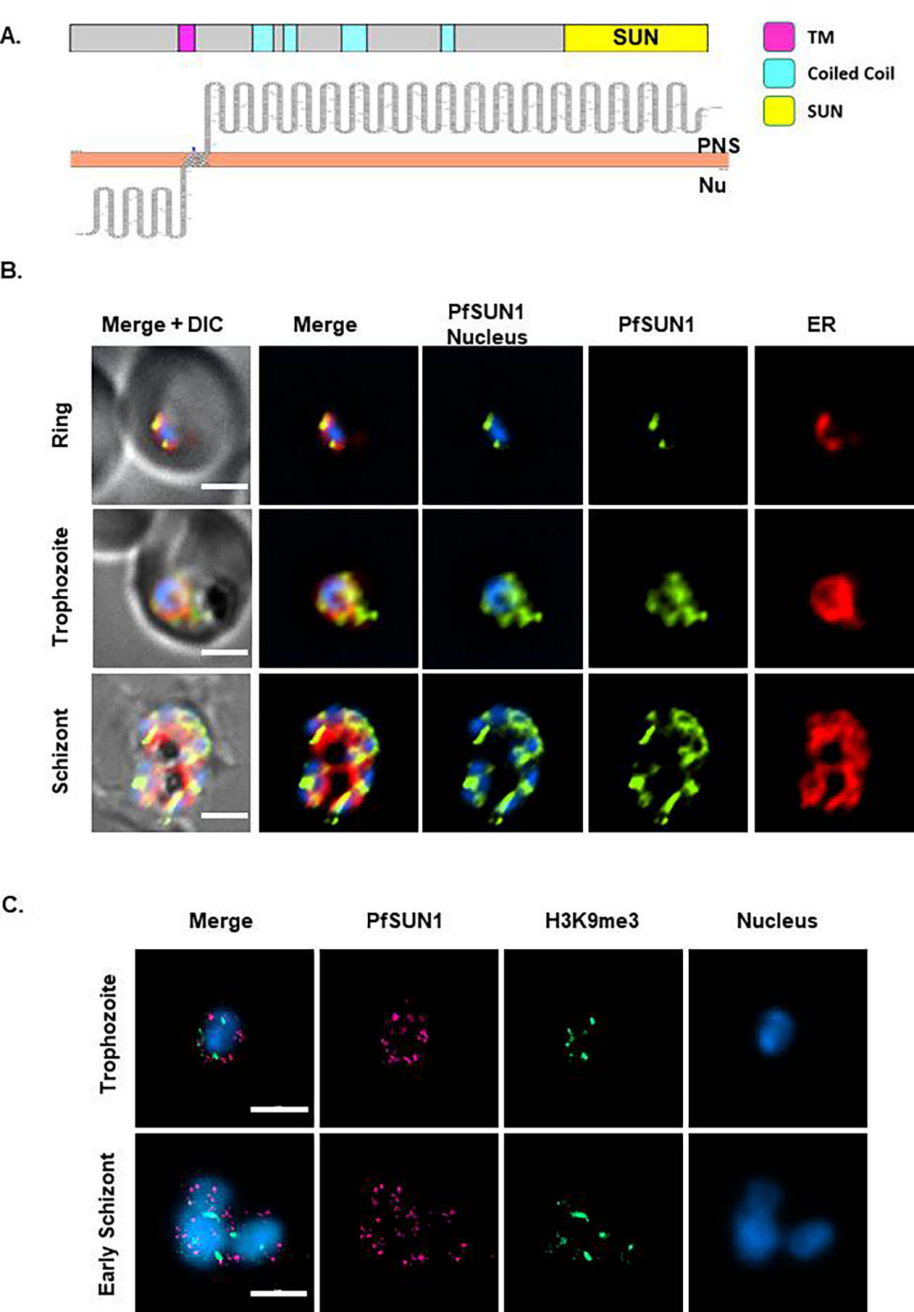
Endogenous PfSUN1 is not associated with heterochromatin at the nuclear periphery. (A) Schematic representation of domain architecture of PfSUN1 (SUN domain; yellow, TM domain; magenta, coiled-coil domains; cyan), membrane topology prediction was analyzed using Protter (28) (PNS; perinuclear space. Nu; nucleoplasm). (B) IFA imaging demonstrating the expression of endogenous PfSUN1-HA during different stages of IDC in PfSUN1-HA-*glmS* transgenic parasites. PfSUN1-HA (green), ER marker (BiP, red), and nuclei were stained with DAPI (blue). Scale bar: 2 µm. (C) dSORTM imaging of endogenous HA-tagged PfSUN1 (magenta) along with heterochromatin histone mark H3K9me3 (green). Nuclei were stained with YOYO1 stain (blue). Scale bar: 2 µm.

**Fig 4:**
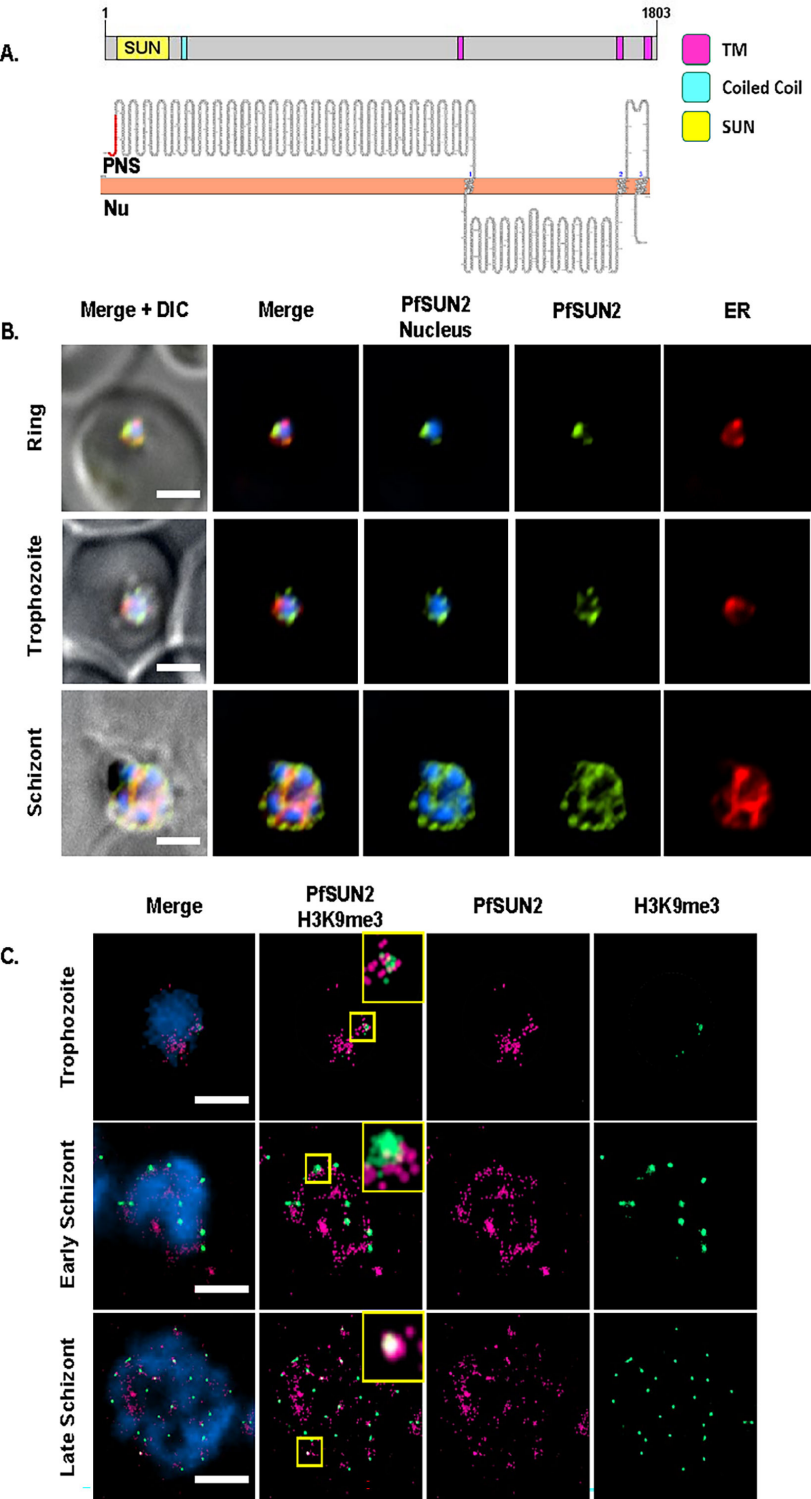
PfSUN2 is an NE protein associated with heterochromatin. (A) Schematic representation of domain architecture of PfSUN2 (SUN domain [yellow]; TM domain [magenta]; coiled-coil domains [cyan]). Membrane topology prediction was analyzed using Protter (28) (PNS, perinuclear space; Nu, nucleoplasm). (B) IFA imaging demonstrating the distribution of endogenous PfSUN2-HA during different stages of IDC in PfSUN2-HA-*glmS* transgenic parasites. PfSUN2-HA (green), ER marker (BiP, red), and nuclei were stained with DAPI (blue). Scale bar: 2 µm. (C) dSTORM imaging of endogenous HA-tagged PfSUN2 (magenta) along with heterochromatin histone mark H3K9me3 (green). Nuclei were stained with YOYO1 (blue). Scale bar: 2 µm. insert: higher magnification of marked foci.

**Fig 5:**
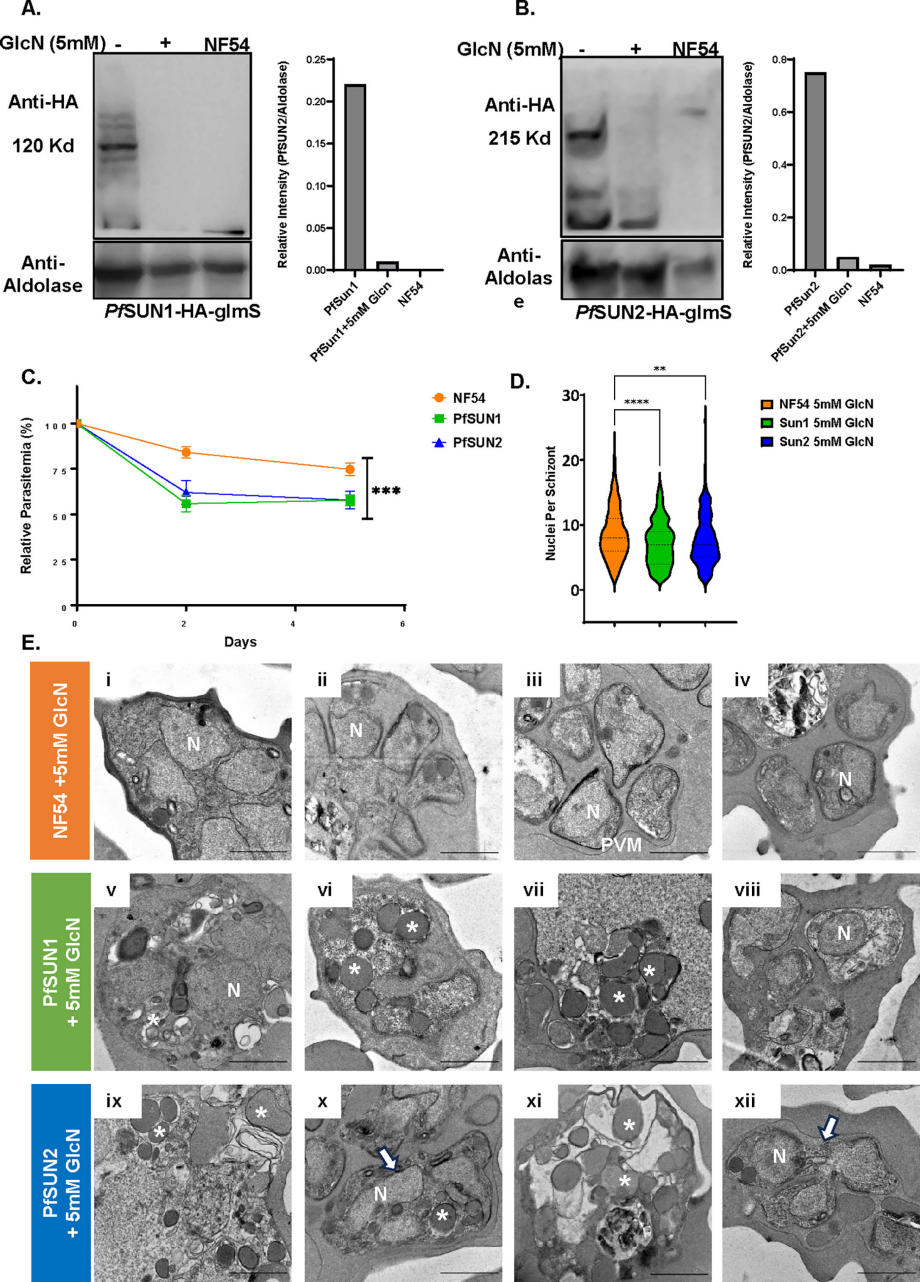
PfSUN1 and PfSUN2 expression is essential for the proper proliferation of blood-stage parasites. (A) Inducible knockdown of endogenous PfSUN1. Western blot (WB) analysis demonstrating inducible knockdown by growing PfSUN1-HA-*glmS* parasites in the presence or absence of 5 mM GlcN for 72 h. PfSUN1 was detected using an anti-HA antibody, while an anti-aldolase antibody was used as a loading control. (B) Inducible knockdown of endogenous PfSUN2. WB analysis of PfSUN2-HA-*glmS* parasites grown in the presence or absence of 5 mM GlcN over a 72 h time course. PfSUN2 was detected using anti-HA antibody, while anti-aldolase antibody was used as a loading control. (C) Inducible knockdown of PfSUN1 and PfSUN2 causes significant growth delay. PfSUN1-HA-*glms*, PfSUN2-HA-*glms*, or NF54 WT parasites were grown either in the presence or absence of 5 mM GlcN, with parasitemia determined daily by flow cytometry. Growth delays are expressed as the ratio of parasitemia at each time point between the GlcN treated and untreated parasites for each of the three lines. Experiments were performed in biological triplicates (*n* = 3). Statistical significances between different stages were determined using Student’s *t*-test (***P < 0.001). (D) Violin plots show the distribution of nuclei per schizont, with the median (thick dashed line), and 25% and 75% quartile ranges indicated (thin dashed lines) for NF54, PfSUN1, and PfSUN2 lines treated with 5 mM GlcN. Data from Giemsa-stained blood smears of tightly synchronized parasites of each line used for EM were obtained from 200–300 schizonts and counted independently by two observers. Statistical significance was determined using pairwise comparisons; **P < 0.01 and ****P < 0.0001. (E) Ultrastructure analysis using transmission electron microscopy of schizonts grown in the presence of 5 mM GlcN. NF54 wild-type parasites showing normal morphology in pre-segmentation (i), mid-segmentation (ii), PVM rupture (iii and iv), PfSUN1-HA-*glmS* (v–viii), and PfSUN2-HA-*glmS* parasites (ix–xii) grown on GlcN for 96 h to induce complete knockdown of PfSUN1 and PfSUN2. Representative micrographs showing abnormal morphology of pre- and post-segmented schizonts with numerous membrane-bound vesicles in their cytoplasm, appearance of electron-dense membrane-bound structures, loss of internal structures, and impaired segmentation. Asterisks indicate vacuolar structure, and arrows point to unsegmented nuclei. Scale bar: 1 µm. N, nucleus; PVM, parasitophorous vacuole.

To better understand the role of the two putative SUN-domain proteins in *P. falciparum*, we used the pSLI system (27) to create transgenic parasite lines in which PfSUN1 or PfSUN2 are endogenously tagged with an hemagglutinin (HA) epitope and fused to the *glmS* ribozyme that allows one to perform conditional knock-down by adding glucosamine (GlcN) to the culture media. These lines were termed PfSUN1-HA-*glmS* and PfSUN2-HA-*glmS*, respectively. Following the isolation of a clonal transgenic population and confirming the correct integration into the genome (Fig. S4 and 5 S4), we imaged their cellular localization to confirm that the endogenously tagged PfSUN1 localized to the nuclear periphery. We initially performed immunofluorescence assay (IFA) using anti-BiP as a marker for the endoplasmic reticulum (ER) that in *P. falciparum* appears to be surrounding the nuclear staining. Indeed, we found that PfSUN1 localizes to the nuclear periphery with no complete overlap with the ER. To gain more accurate imaging of PfSUN1 and overcome the diffraction limits of light microscopy, we used dSTORM imaging to confirm that the endogenously tagged PfSUN1 localized to the nuclear periphery (Fig. 3A and B) and was not associated with the heterochromatin marker H3K9me3 (Fig. 3C) as observed in its ectopic expression. Similar results were obtained for the endogenously tagged PfSUN2 (Fig. 4). PfSUN2 is expressed at the nuclear periphery during the IDC, demonstrating regions of a stronger signal where the protein appears to be accumulated (Fig. 4B and C). Interestingly, our dSTORM imaging data suggest that the foci of PfSUN2 are associated with foci of heterochromatin (marked by H3K9me3 antibody) at the nuclear periphery (Fig. 4C). Using 5 mM GlcN, we were able to downregulate the expression of PfSUN1 and PfSUN2 (Fig. 5A and B) and show that this knockdown decreased the proliferation rate of the cultured parasite population (Fig. S6A and B). We normalize the basal growth rate of each line and the effect of GlcN on growth by calculating the ratio of parasitemia of each line with or without GlcN and show that knockdown of both PfSUN1 and PfSUN2 causes a significant growth delay (Fig. 5C), associated with a decrease in the number of nuclei per schizont and an increase in the number of parasites with aberrant nuclei segregation (Fig. 5D and Fig. S10). Altogether, these data demonstrate that PfSUN1 and PfSUN2 are NE proteins, which are essential for the proper proliferation of *P. falciparum*.

### Depletion of PfSUN1 and PfSUN2 results in morphological alterations of the parasites

SUN-domain proteins were shown to play an important role in maintaining nuclear architecture (29). We hypothesized that the reduction in growth rates of parasite populations in which PfSUN1 and PfSUN2 expression was knocked down could imply that some of these parasites are unable to segregate and produce viable daughter merozoites. To better understand if this growth phenotype and its associated morphological changes observed by Giemsa stains (Fig. S10), we used transmission electron microscopy (TEM) to evaluate the ultrastructural changes in the parasites’ morphology during replication. We found that while NF54 parasites appear to be able to divide their nuclei and segregate properly (Fig. 5E, upper panel), significant morphological deformations and loss of internal structures are observed during the progression of schizogony in many (but not all) of the parasites in which PfSUN1 (Fig. 5E, middle panel) and PfSUN2 (Fig. 5E, lower panel) are downregulated. Large electron-dense membrane-bound vacuolar structures could be observed in these parasite populations, which may represent an apoptotic-like phenotype of parasites that will not be able to complete their intraerythrocytic development. These data indicate that SUN-domain proteins in *P. falciparum* play a role in proper nuclei segregation and cellular division into daughter merozoites during schizogony.

**Fig 6:**
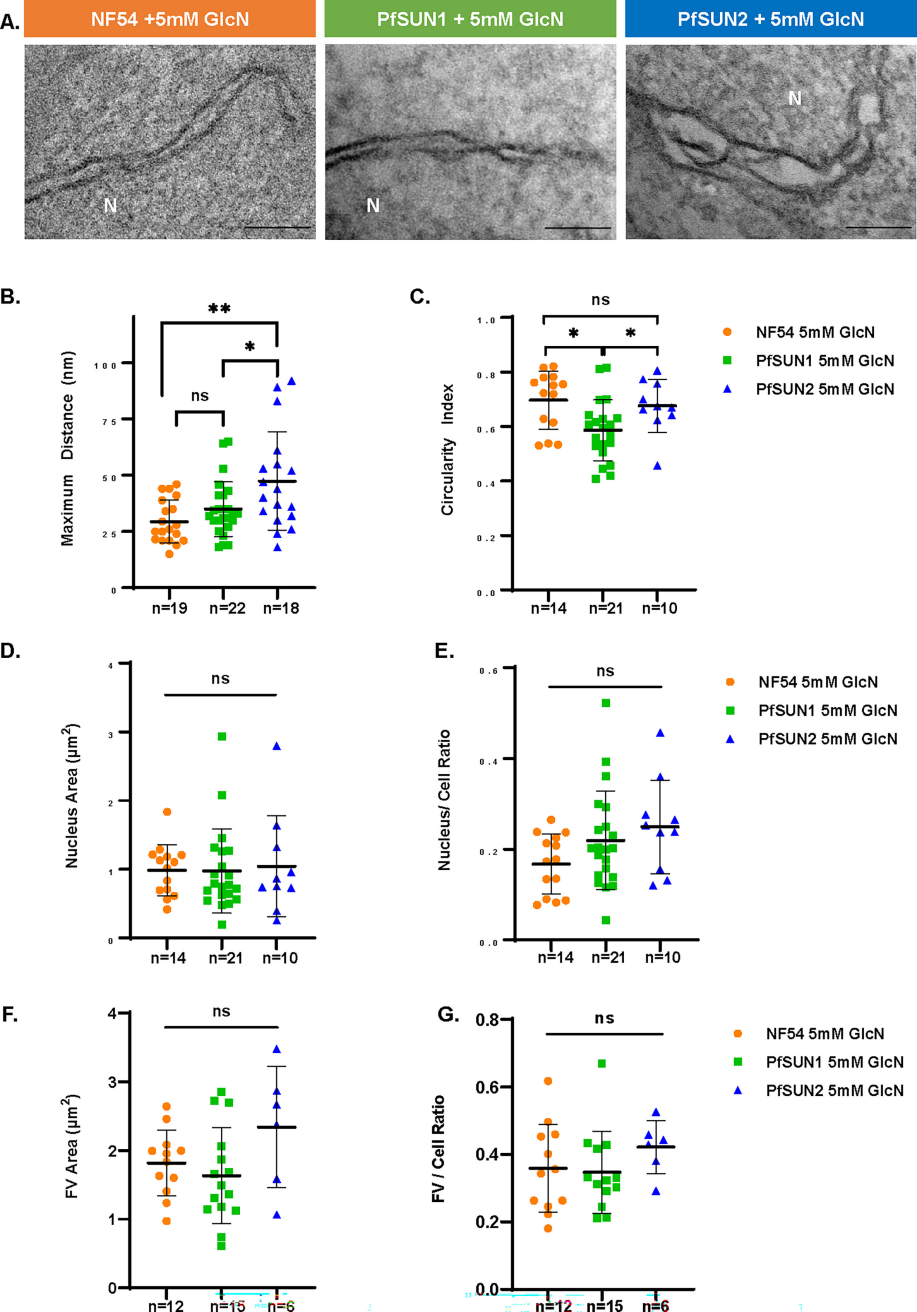
Loss of PfSUN1 and PfSUN2 expression impairs NE homeostasis. Ultrastructural analysis of the nuclear and cellular morphology of late trophozoite of PfSUN1-HA-*glmS* and PfSUN2-HA-*glmS* parasites grown on 5 mM GlcN for 82 h was examined using TEM. NF54 wild-type parasites were grown in parallel on 5 mM GlcN for 82 h as a control. (**A**) Representative electron tomography images showing NE expansion in PfSUN2 inducible knockdown line comrade to NF54 and PfSUN1 inducible knockdown. N: nucleus. Scale bar: 100 nm. (**B**) Comparison of the maximum distance between the INM and ONM measured for the three lines. (**C**) Comparison of the circularity index of trophozoite of the three lines. (**D**) Comparison of the distribution of nuclear area (µm²). (**E**) Nuclear area to cell area ratio of the three lines. (**F**) Comparison of the distribution of FV area (µm²). (**G**) FV area to cell area ratio of the three lines. All measurements and morphometric analysis were performed using the measure feature in ImageJ. Error bars represent SD. Statistical significances between different groups were determined using an unpaired Student’s *t*-test (***P < 0.001, **P < 0.01, *P < 0.05, and ns; *P* > 0.05).

**Fig 7:**
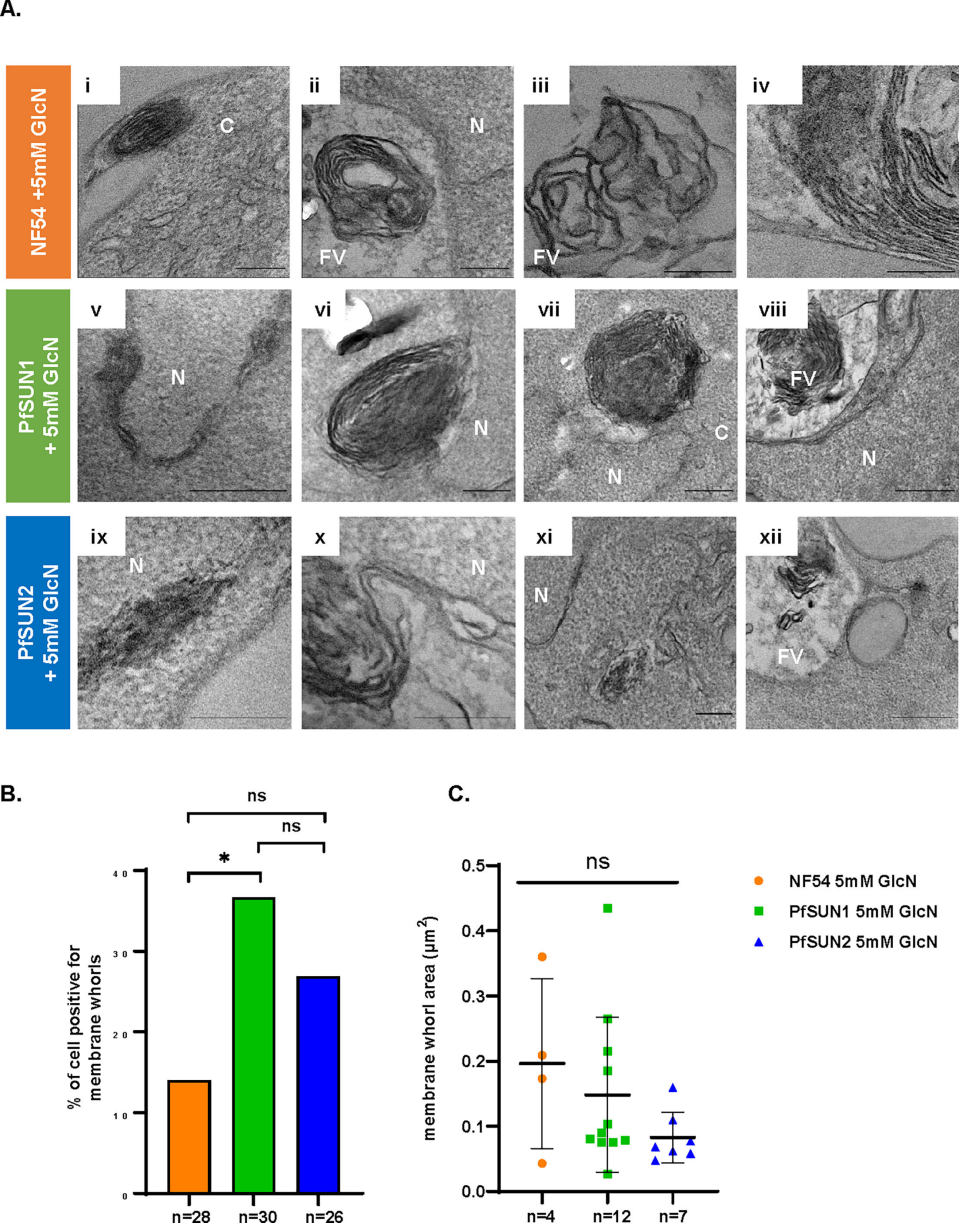
Loss of PfSUN1 and PfSUN2 expression results in nuclear membrane expansion and membrane whorls formation. (A) Ultrastructure analysis of the late trophozoite stage of PfSUN1-HA-*glmS* and PfSUN2-HA-*glmS* grown on 5 mM GlcN for 82 h was performed using TEM. NF54 wild-type parasites grown on 5 mM GlcN for 82 h were used as control (i–iv). Representative TEM images showing the expansion of the nuclear membrane and subsequent formation of membranous whorls, that are later observed in the FV, following inducible knockdown of PfSUN1 (v–viii) and PfSUN2 (ix–xii). (B) Membrane whorls abundance and (C) area distribution (µm²) in each of the three lines. All measurements were performed using the measure feature in ImageJ. Error bars represent SD. Statistical significances between different groups were determined using an unpaired Student’s *t*-test (***P < 0.001, **P < 0.01, *P < 0.05, and ns; *P* > 0.05). Scale bars: i, ii, iii, v, vi, vii, ix, x, and xi 200 nm; iv, viii, and xii 500 nm.

As part of the LINC complex, SUN-domain proteins that are embedded in the NE could play a role as a scaffold that holds the INM and ONM of the NE. We, therefore, investigated whether depletion of PfSUN1 and PfSUN2 would affect the morphology of the NE. To this end, we used TEM on tightly synchronized trophozoites and were able to detect numerous deformations in the NE of parasites in which PfSUN2 expression is knocked down (Fig. 6A and B). However, the NE of the PFSUN1 knockdown parasite appeared to have normal morphology. In particular, we found that the average measured distance between the INM and the ONM was significantly wider in the PfSUN2 knockdown parasites. On the other hand, we found that PfSUN1 knockdown significantly reduced nuclei circularity, while PfSUN2 showed no significant impact (Fig. 6C). We also measured additional morphological parameters such as nuclei size, nuclei/cell ratio, food vacuole (FV) size, and the ratio between the size of the food vacuole and the entire cell. In any of these parameters, we were unable to detect significant change resulting from PfSUN1 and PfSUN2 knockdown (Fig. 6D through G). Interestingly, in both PfSUN1 and PfSUN2 knockdown parasites, we observed duplication of the NE membranes (Fig. 7A, v and ix, and Fig. S8A and S9A, respectively). In addition, we observed the formation of concentric lamellar membranous whorls that involve both the INM and ONM, and in some parasites, these whorls appeared to be budding from the NE (Fig. 7A, vi and x, and Fig. S8B and S9B, respectively), while they were also observed in the cytoplasm (Fig. 7A, vii and xi, and Fig. S8C and S9C, respectively) or the FV (Fig. 7A, viii and xii, and Fig. S8D and S9D, respectively). Such membranous structures were also observed in NF54 parasites (Fig. 7A, i–iv, and Fig. S7); however, their abundance was more pronounced following PfSUN1 knockdown, with 40% of the parasites featuring them, as compared to 27% in the PfSUN2 knockdown and only 14% of the NF54 parasites (Fig. 7B). In addition, we observed that the area of the membranous whorls in our thin sections varied between parasites (Fig. 7C).

### PfSUN1 is essential for activating the DNA damage response

**Fig 8:**
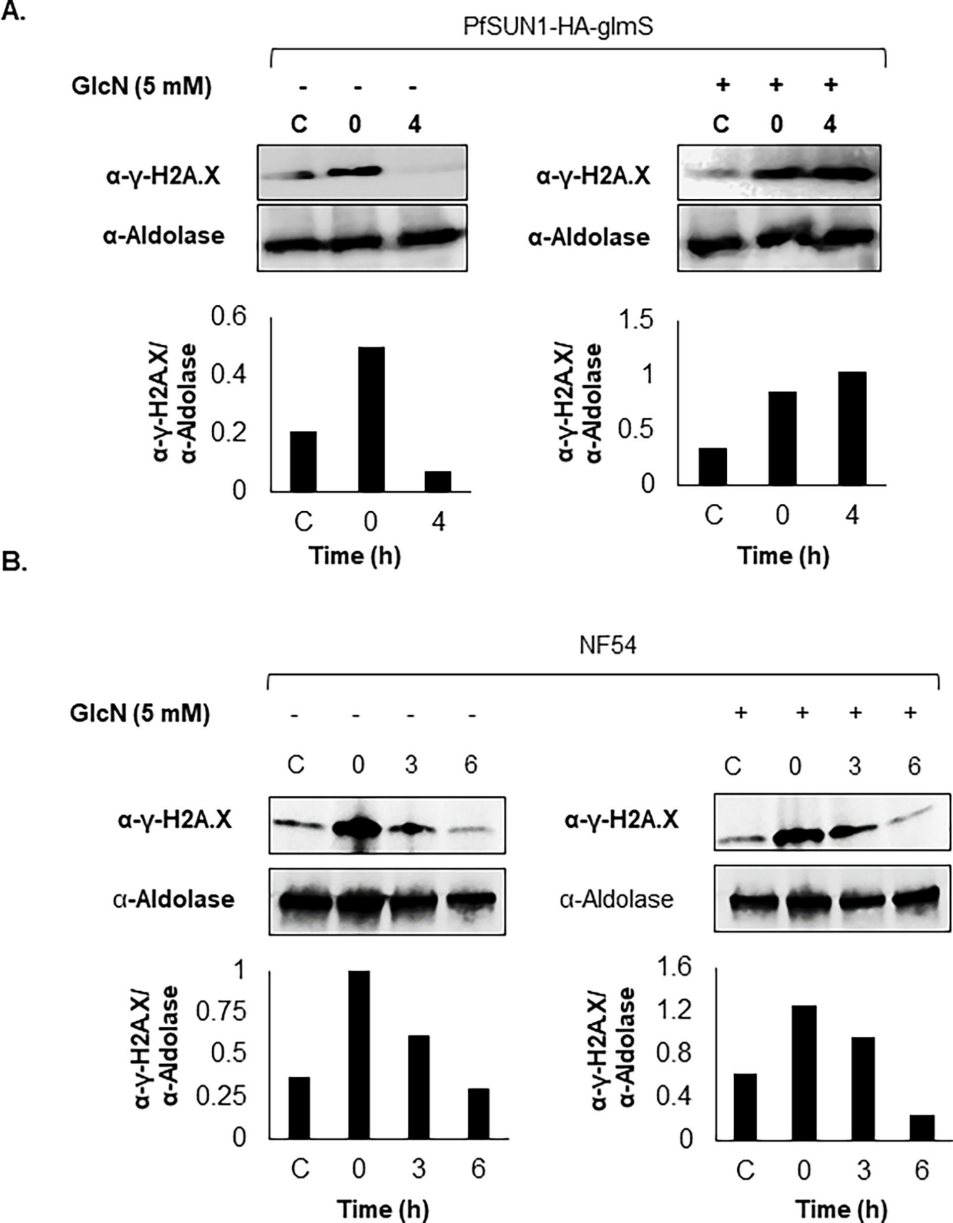
PfSUN1 is essential for activating the DDR. (A) The role of PfSUN1 and PfSUN2 in DNA damage repair by irradiating PfSUN1-HA-*glmS* and PfSUN2-HA-*glmS* parasites which were grown for 72 h either on regular media (left) or on media supplemented with 5 mM GlcN (right). Tightly synchronized ring stages were exposed to a sub-lethal dose of X-ray irradiation (6,000 rad). (Upper panel) Dynamics in phosphorylation of PfH2A measured by WB analysis of protein extracted from PfSUN1-HA-*glmS* parasites before and after irradiation in the presence (left) or absence (right) of PfSUN1 expression. C, control untreated; 0, 15 min after X-ray irradiation; 4, 4 h after irradiation. Activation of DDR and subsequent repair was analyzed using anti-γ-PfH2A.X antibody. Anti-aldolase antibody was used for loading control. (Lower panel) Densitometry quantification of the ratio between the WB signals obtained for γ-PfH2A.X and aldolase presented above. Densitometry analysis was performed using ImageJ software. (B) A similar experiment was performed on NF54 parasites grown for 72 h either on regular media (left) or on media supplemented with 5 mM GlcN (right). Tightly synchronized ring stages were exposed to a sub-lethal dose of X-ray irradiation (6,000 rad). (Upper panel) Dynamic in phosphorylation of PfH2A measured by WB analysis of protein extracted from control (untreated) and NF54 parasites before and after irradiation in the presence (right) or absence (left) of GlcN. C, control untreated; 0, 15 min after X-ray irradiation; 3, 3 h after irradiation; 6, 6 h after irradiation. Activation of DDR and subsequent repair was analyzed using anti-γ-PfH2A.X antibody. Anti-aldolase antibody was used for loading control. (Lower panel) Densitometry quantification of the ratio between the WB signals obtained for γ-PfH2A.X and aldolase presented above. Densitometry analysis was performed using ImageJ software.

Previous work in mammalian cells showed that the mouse SUN-domain proteins SUN1 and SUN2 play an important role in the DDR (30). Malaria parasites replicate their haploid genome multiple times through consecutive mitotic cycles and are particularly prone to error during DNA replication. Therefore, we were interested to determine whether PfSUN1 and PfSUN2 play a role in the *P. falciparum* DDR. To this end, we applied a DDR assay which was recently optimized for *Plasmodium* spp*.* and uses anti-γ-H2A.X antibody that recognizes the phosphorylated PfH2A, which serves as a marker for DNA damage in *P. falciparum* (31). The two transgenic lines, PfSUN1-HA-*glmS* and PfSUN2-HA-*glmS*, were exposed to a sub-lethal dose of X-ray irradiation (3,000 rad), and the kinetics of PfH2A phosphorylation were followed in the presence or absence of PfSUN1 expression (Fig. 8A). We found that the PfSUN1-HA-*glmS* parasites, which were grown without GlcN, showed a decrease in the phosphorylation level of PfH2A over time (4 h after irradiation), corresponding with the repair of DNA damage caused by irradiation (Fig. 8A, left panels). However, parasites grown on GlcN for 72 h prior to the X-ray irradiation were unable to repair the DNA damage, and the levels of PfH2A phosphorylation remained constant over time (Fig. 8A, right panels). Nonetheless, we show that GlcN had no effect on the ability of NF54 parasites to activate DDR (Fig. 8B). We were unable to detect any difference in DNA damage repair in the presence or absence of PfSUN2 (data not shown).

## DISCUSSION

The complex biology of *Plasmodium* parasites and particularly their unique replication by closed mitosis during schizogony requires special adaptations of their nuclear structure and functions that enable them to proliferate and thrive in different cellular niches within their hosts. Similar to higher eukaryotes, specific nuclear functions in *Plasmodium* were found to be linked to different sub-nuclear compartments. A marked example is their nuclear periphery, a sub-nuclear compartment that was implicated in the regulation of gene expression, particularly of genes involved in virulence and immune evasion (3). Nonetheless, since only a few components of the nuclear envelope have been identified, the nuclear periphery of *Plasmodium* refers primarily to the margins of the DNA staining imaged by light microscopy, while the precise morphological determination of this sub-nuclear compartment remains elusive. In addition, the role of the LINC complex that links the nuclear periphery with the cytoplasm remained understudied in these parasites, despite its importance for diverse nuclear functions, including nuclear replication and positioning, movement of chromosomes, and DNA repair. Moreover, in model eukaryotes, some of the nuclear dynamics required for replication and chromosome movement were shown to involve interactions between the LINC complex and lamins (29) that serve as the nuclear scaffold below the inner nuclear membrane. Thus, in an organism such as *Plasmodium* that lacks conventional lamins, the mechanisms by which the LINC complex could mediate such nuclear dynamics remain a mystery, similar to African trypanosomes that do not encode for conventional lamins or canonical components of the LINC complex (32). SUN-domain proteins are major components of the LINC complex with many organisms expressing different SUN-domain variants in a cell cycle and in a tissue-specific manner (33). One example is the SUN-domain protein (TgSLP1) of the apicomplexan parasite *Toxoplasma gondii* which showed stage-specific expression and was found to be essential for nuclear division and centrocone integrity (34). Interestingly, the two SUN-domain proteins, which we found to be essential for the proper proliferation of blood-stage *P. falciparum* parasites, have different temporal transcription profiles during the IDC. It appears that *pfsun1* transcripts are highly abundant in male gametocytes and ookinetes and transcript levels of *pfsun2* peak in mature liver and blood-stage merozoites (35). These differential patterns may imply that in addition to their roles in the IDC, they could also be important for parasite-specific functions in other stages of the parasite life cycle. A recent systematic screening for *Plasmodium* fertility genes identified *P. berghei* SUN1 complex and demonstrated its essentiality for male gamete fertility by linking the MTOC to the nuclear envelope and enabling mitotic spindle formation during male gametogenesis (21).

While both PfSUN1 and PfSUN2 are important for parasite proliferation during the IDC, each could have specific functions as it appears that PfSUN2 is more tightly associated with heterochromatin, while PfSUN1 plays a specific role in the DNA damage response. We have previously demonstrated that the *Plasmodium* NE is actively remodeled during asexual replication (25), changing the number, organization, and orientation of the NPCs. Interestingly, the NPC clusters change their directionality as mitosis progresses, indicating the possible rotation of the nucleus, which in some cases reaches up to 180° during late schizogony (25). Remarkably, during this rotation, the cluster of the NPC maintains its orientation facing the parasite’s membrane, providing evidence for possible cytoplasmic inﬂuence on NPC localization during the transition from a multinucleated syncytium to multiple autonomous cells. Interestingly, we observed similar directionality in the localization of both PfSUN1 and PfSUN2 throughout the IDC. PfSUN1 displays a highly polarized localization pattern in mature merozoites just before egress that is reminiscent of the previously observed clustering of NPCs at this stage. However, even though the NPC cluster at that stage is found adjacent to the foci of PfSUN1, they do not completely co-localize at any of the asexual stages, similar to what was observed in the fission yeast *Saccharomyces cerevisiae*, where the SUN-domain protein MPS3 does not co-localize with the NPC (36). Nonetheless, it was shown that the disruption of the LINC complex, as a critical component of force transmission between the nucleus and cytoskeleton, caused impaired nuclear positioning and cell polarization (37, 38). The dynamic nuclear positioning and the polarity of NPC clusters during plasmodium schizogony appear to be associated with the polarity of PfSUN1/2 accumulation observed here. Their cell-cycle-dependent polarity, along with the slow growth rate and abnormal schizont morphology of parasites in which PfSUN1/2 was knocked down, strongly suggests that PfSUN proteins play an important role in nuclear division and cellular segregation during schizogony. In the rodent malaria parasite *P. berghei*, PbSUN1 displayed a polarized localization near spindle poles, where it facilitates rapid genome replication and NE remodeling in male gametocytes (22). These findings align with the polarity of PfSUN1 in segregating nuclei during asexual schizogony in *P. falciparum*, highlighting a possible conserved role across apicomplexan in coordinating nuclear-cytoskeletal interactions. Interestingly, *Cryptosporidium* spp. encode for orthologs of LINC complex components, which could also be implicated in its complex division processes that include synchronized nuclear replication and membrane remodeling during sexual and asexual stages; however, this hypothesis requires further experimental evidence (39)

In addition to their impaired ability to segregate properly and the observed morphological alteration of the perinuclear space, the most striking morphological phenotype was the appearance of the membrane whorls, which was more significant following PfSUN1 knockdown. These membrane whorls appeared to be forming and pinching out at a distinct location at the NE and, in many cases, were finally observed at the parasite food vacuole—presumably for lysosomal-like degradation (40, 41). This could represent a sequential process that is reminiscent of microautophagy described in yeast, where ER whorls are translocated to the lysosome for degradation in mechanisms that are similar to known autophagy machinery (42). SUN-domain proteins were previously implicated in homeostasis of the nuclear envelope in *S. cerevisiae*, where mutations in the SUN protein MPS3 led to over proliferation of the NE (43), similar to what we observed here. It is interesting to note that the depletion of SUN1 in *P. berghei* led to significant changes in lipid metabolism, linking NE dynamics with broader cellular processes such as membrane biogenesis and metabolic regulation (22). In addition, SUN proteins were shown to play a role in remodeling the NE during open mitosis by facilitating membrane removal from chromatin through NE breakdown (44). Evidently, disassembly of the LINC complex is required to control nuclear envelope dynamics during ER stress to allow specific autophagy responses that maintain the homeostasis of the NE and the ER (45). Altogether, these data suggest that SUN proteins in *P. falciparum* may play a role in the homeostasis of the NE and the ER.

In addition to the abnormal replication phenotype, we found that PfSUN1 is essential for activating the DDR. In recent years, there is mounting evidence that the LINC complex plays an important role in different aspects of the DDR (15). Recently, we showed that a plasmodium bona fide SR protein, PfSR1, is essential for DNA repair that takes place at specific foci at the nuclear periphery (46). We found that after exposing the parasite to a source of damage, PfSR1 is recruited to the site of damage where it co-localizes with PfRAD51. Furthermore, we demonstrated that PfSR1 expression is essential for the recruitment of PfRAD51 to the site of damage as well as for the parasite’s ability to activate DDR. These data suggest that the nuclear periphery of *P. falciparum* contains functional sub-compartments specific for DNA repair. In *Caenorhabditis elegans* as well as in mammalian cells, SUN-domain proteins were shown to be involved in the recruitment of DNA repair proteins such as DNA-dependent protein kinase, Rad51, Ku70/Ku80, and FAN1 nuclease to the site of damage. In addition, SUN-domain proteins can influence which repair mechanisms will be applied by promoting homologous recombination (HR) over nonhomologous end joining (NHEJ) (14). Damaged DNA must be mobile in the nucleoplasm to be directed and reach the site of repair at the nuclear periphery. It was shown that chromatin surrounding a double-strand break has greater mobility which is mediated by cytoplasmic microtubules that interact with nucleoplasmic 53BP1 through required interaction with SUN-domain proteins (13). Although the exact mechanistic role of components of the LINC complex in DDR remains elusive, it is reasonable to suggest that SUN-domain proteins in plasmodium could contribute to the formation of a functional repair site at the nuclear periphery and mobilize both the damaged locus and components of the repair machinery to create the sub-nuclear repair site.

Our study provides the first glimpse into the role of SUN proteins as possible components of the *Plasmodium* LINC complex essential for various nuclear functions in blood-stage parasites. The identification of PfSUN proteins could further be exploited for the discovery of other components of the LINC complex and the nuclear envelope. In addition, this knowledge could be further utilized to investigate their involvement in the dynamics of gene positioning at the nuclear periphery, interactions, and movement of chromosomes and anchoring specific loci to functional sub-nuclear sites.

## MATERIALS AND METHODS

### Parasite culturing and parasitemia counts

Parasites were kept in continuous culture as in reference 47. All parasites used were derivatives of the NF54 parasite line and were cultivated at 5% hematocrit in RPMI 1640 medium, 0.5% Albumax II (Invitrogen), 0.25% sodium bicarbonate, and 0.1 mg/mL gentamicin. Parasites were incubated at 37°C in an atmosphere of 5% oxygen, 5% carbon dioxide, and 90% nitrogen. Depending on the required experiments, parasitemia (percentage of infected red blood cells [iRBCs] out of the total red blood cells [RBCs]) was kept at 0.1%–5% by dilution, and the medium was changed at least every second day. Parasite cultures were synchronized using percoll/sorbitol gradient centrifugation as previously described (48, 49). Briefly, infected RBCs were layered on a step gradient of 40%/70% percoll containing 6% sorbitol. The gradients were then centrifuged at 12,000 g for 20 min at room temperature. Highly synchronized, late-stage parasites were recovered from the 40%/70% interphase, washed twice with complete culture media, and placed back in culture with an adjusted hematocrit of 5%. Parasite cultures were synchronized using sorbitol as previously described (50). Briefly, the culture was transferred into a 15 mL falcon tube and spun down at 1,800 × *g* for 3 min. The supernatant was discarded, and the pellet was resuspended in a pre-warmed 5% D-sorbitol solution. After incubation for 10 min at 37°C, the falcon tube was vortexed for 5 seconds and centrifuged at 800 g for 5 min. The pellet was washed with RPMI complete medium and re-cultured in a fresh RPMI complete medium. The hematocrit was adjusted to 5%. Sorbitol synchronization leads to a culture holding only ring-stage parasites. Transgenic parasites were cultured in the presence of drugs corresponding to the respective selectable marker used. The level of parasitemia was calculated either by flow cytometry. For flow cytometry, aliquots of 50 µL parasite cultures were washed in phosphate-buffered saline (PBS) and incubated for 30 min with 1:10,000 SYBR Green I DNA stain (Life Technologies). The fluorescence profiles of infected erythrocytes were measured on CytoFLEX (Beckman Coulter) and analyzed by the CytExpert software.

### Parasite transfection

Parasites were transfected as described (51). Briefly, 0.2 cm electroporation cuvettes were loaded with 0.175 mL of erythrocytes and 50–100 µg of purified plasmid DNA in incomplete cytomix (8.95 g KCl, 0.017 g CaCl, 0.76 g EGTA, 1.02 g MgC_2_, 0.871 g K_2_HPO_4_, 0.68 g KH_2_PO_4_, and 7.08 g HEPES) solution. Electroporation was performed using the Gene Pulser Xcell (Bio-Rad, 310 V, 950 µF, ∞ Ω). Electrophorized blood was washed twice in a complete RPMI medium and transferred to a fresh culture flask with 10 mL of fresh complete RPMI, and schizonts were isolated using percoll/sorbitol synchronization. The blood was then adjusted to a hematocrit of 5%. After 24 h, the selection drug was added, and the medium was changed daily for the first 5 days and every second day from that day onward. Stable transfectants carrying plasmids with an human dihydrofolate reductase (hDHFR)-selectable marker were selected on 4 nM WR99210, and those carrying yeast dihydroorotate dehydrogenase (yDHODH) were selected on 1.5 µM DSM1. Stable transfectants carrying plasmids with blasticidin S deaminase (BSD)-selectable marker were initially selected on 2 µg/mL blasticidin-S (Invitrogen). In order to obtain parasites carrying large plasmid copy numbers, these cultures were then subjected to elevated concentrations of 6–10 µg/mL blasticidin-S, depending on the experimental icidin design.

### Bioinformatics analyses

In order to find putative SUN-domain proteins in *P. falciparum*, a BLAST search was performed against the SUN domain annotated sequence of human SUN-domain proteins in the Plasmodium database PlasmoDB (52). Illustrations of domain architecture were generated using Illustrator for Biological Sequences (IBS) illustrator (53). The SUN-domain sequences of selected SUN-domain proteins were obtained from UniProt (54). Membrane topology prediction was analyzed using Protter (28). Multiple-sequence alignment of PfSUN1 and PfSUN2 (PF3D7_1215100 and PF3D7_1439300) was performed using CLUSTALO (55) and further analyzed using the ESPript3 program (56). A homology model of the SUN domain of PfSUN1 and PfSUN2 was built by comparative modeling using the crystal structure of HsSUN2 (Protein Data Bank entry 4DXT) by using the I-TASSER server (57). The structure visualization of the PfSUN1 and PfSUN2 three-dimensional model was performed using the PyMOL program (58). Gene expression profiles were analyzed using resources available in the PlasmoDB database, derived from previously published RNA-seq data sets. The data sets were pre-normalized to transcripts per million (TPM) by the original authors, allowing for direct comparison. For the IDC, only well-annotated data points were included to ensure accuracy. *P. berghei* data were used for liver stages due to the lack of *P. falciparum* data, enabling a complete life cycle profile. Mean TPM values were averaged from the combined data sets to provide a reliable representation of gene expression (59–70).

### Immunofluorescence assay

IFA was performed as described (31). In brief, iRBCs were washed twice with PBS and resuspended in a freshly prepared fixative solution, 4% paraformaldehyde (EMS; Electron Microscopy Sciences), and 0.0075% glutaraldehyde (EMS) in PBS, for 30 min at room temperature. Following fixation, iRBCs were permeabilized with 0.1% Triton X-100 (Sigma) in PBS and then blocked with 3% bovine serum albumin (BSA; Sigma) in PBS. Alternatively, parasites were released from iRBCs with saponin, washed, and fixed in 4% PFA on ice for 1 h. Cells were then incubated with the following primary antibodies, used at the indicated dilutions: mouse anti-GFP (Roche 1:300), mouse anti-HA (Roche:100), rabbit anti-myc (Cell Signaling 1:100), for ER staining rabbit anti-BiP (MR4 1246, 1:200) for 1.5 h at room temperature, and washed three times in PBS. Following this, cells were incubated with the following secondary antibodies conjugated to Alexa fluorophores: Alexa Fluor 488 goat anti-mouse, Alexa Fluor 568 goat anti-mouse, and Alexa Fluor 568 goat anti-rabbit (Life Technologies; 1:500) antibodies for 1 h at room temperature. Cells were washed three times in PBS and laid on polytetrafluoroethylene printed slides (EMS) and mounted in ProLong Gold antifade reagent with DAPI (Molecular Probes). Fluorescent images were obtained using a Plan Apo λ 100 × oil immersion lens (numerical aperture [NA]  = 1.5; working distance [WD]  = 130 µm) on a Nikon Eclipse Ti-E microscope equipped with a CoolSNAPMyo CCD camera. Images were processed using NIS-Elements AR (4.40 version) software.

### Immunogold labeling and electron microscopy analysis

Cells were fixed in 4% paraformaldehyde with 0.1% glutaraldehyde in 0.1M cacodylate buffer (pH  =  7.4) for 1 h at room temperature and kept overnight at 4°. The samples were soaked overnight in 2.3 M sucrose and rapidly frozen in liquid nitrogen. Frozen ultrathin (70–90 nm) sections were cut with a diamond knife at −120°C on a Leica EM UC6 ultramicrotome. The sections were collected on 200-mesh Formvar-coated nickel grids. Sections were blocked with a solution containing 1% BSA, 0.1% glycine, 0.1% gelatin, and 1% Tween 20. Immuno-labeling was performed using affinity purified anti-GFP antibodies (1:20, Abcam), overnight at 4°C, followed by exposure to goat anti-Rabbit IgG coupled to 10 nm gold particles (1∶20, Jackson ImmunoResearch), for 30 min at room temperature. Contrast staining and embedding were performed as previously described (71). The embedded sections were viewed and photographed with a FEI Tecnai SPIRIT (FEI, Eidhoven, Netherlands) transmission electron microscope operated at 120 kV and equipped with an EAGLE CCD Camera.

### Transmission electron microscopy

iRBCs were pelleted, washed twice with PBS, and ﬁxed using 2% formaldehyde and 2.5% glutaraldehyde in 0.1 M cacodylate buffer, pH 7.4 at room temperature for 2 h, following an overnight incubation at 4°C. The iRBCs were washed and post-ﬁxed with 1% osmium tetroxide in the same buffer for 1 h at room temperature, and the samples were dehydrated in graded ethanol series and embedded in Epon. Thin sections (70–90 nm) were prepared using an Ultracut UCT microtome (Leica). Followed post staining with 2% uranyl acetate and Reynold’s lead citrate and viewed in a Jeol JEM-1400 Plus TEM (Jeol, Tokyo, Japan) operating at 100 kV, equipped with ORIUS SC600 CCD camera (Gatan, Abingdon, United Kingdom), and Gatan Microscopy Suite program (DigitalMicrograph, Gatan, UK). Image processing was performed on the DM3 files using FIJI (72)

### STORM imaging and analysis

Stochastic optical reconstruction microscopy (STORM) imaging was performed as described (73) in brief. Parasite cultures were saponin lysed and washed three times with fresh PBS. Parasites were then re-suspended on ice with fresh fixative solution, 4% paraformaldehyde (EMS) in PBS for 1 h. Fixed parasites were allowed to air dry in an eight-well-chambered cover glass system 1.5H (*in vitro* scientific) for 2–3 h at room temperature and covered with glycine (125 mM glycine in PBS) to quench unreacted aldehyde groups. Samples were washed three times with PBS and permeabilized with 0.1% Triton-X 100 (PBS) for 10 min at room temperature, washed with PBS, and blocked with 3% BSA (IgG free, Sigma) in 0.025% Tx-100/PBS for 1 h at room temperature. Primary antibodies, mouse anti-GFP (1:300, Roche), mouse anti-HA (1:100 Roche), rabbit anti-Halo (1:300, Promega), rabbit anti-H3K9me3 (1:300 Abcam), and rabbit anti-H3K9Ac (1:300 Abcam) were diluted in 3% BSA containing 0.025% Tx-100 in PBS and applied for 1 h at room temperature, followed by five washes in PBS containing 0.025% Tx-100. Secondary antibodies, goat anti-mouse Alexa647, and goat anti-Rabbit Alexa568 (1:500, life technologies) were applied for 1 h in 3% BSA containing 0.025% in PBS at room temperature, followed by five washes with 0.025% Tx-100 in PBS. Parasite nuclei were labeled with YOYO-1 (1:1,000, life technologies) for 20 min at room temperature, followed by three washes with PBS. STORM was performed by a Nikon Eclipse Ti-E microscope with a CFI Apo total internal reflection fluorescence (TIRF) ×100 DIC N2 oil objective (NA 1.49, WD 0.12 mm). Multi-channel calibration was performed prior to data acquisition using fluorescent TetraSpeckTM microspheres 0.1 µm in diameter (Life-technologies, Molecular Probes). Stained cells were placed in Glox-MEA imaging buffer containing 50 mM monoethanolamine (MEA), 10% glucose (D_2_O), and Glox (11.2 mg/mL glucose Oxidase [Sigma] and 1.8 mg/mL catalase [Sigma, C30-500MG]) in dilution buffer (50 mM NaCl and 200 mM Tris in D_2_O). Samples were illuminated by 561 nm and 647 nm excitation lasers in changing intensity over the duration of the imaging sequence (typically, using 50%–100% power). A 488 nm laser was used at 0.5% power to visualize nuclei by TIRF. For each acquisition, 10,000 frames were recorded onto a 256 × 256-pixel region (pixel size 160 nm) of an Andor iXon-897 EMCCD camera. Super-resolution images were reconstructed from a series of at least 5,000 images per channel using the N-STORM analysis module, version 1.1.21 of NIS Elements AR v. 4.40 (Laboratory imaging s.r.o.).

### Plasmid construction

In order to express PfSUN1 fused to a GFP tag at its’ C’ terminus, the genomic sequence of PF3D7_1215100 was amplified using PfSUN1-F 5′-AACTGCAGAAAAATGAACATAAGCAACAGT-3′ and PfSUN1-R 5′-GAAGATCTAAATTTTCTTATACATCTTTTT-3′ and cloned into the pHTIDH expression vector (24) using PstI and BglII to generate pHTIDH-SUN1GFP. In order to express PfSec13 fused to a Halo tag, the genomic sequence of Pf3D7_1230700 was amplified by using the primers Sec13Halo-F GCGATCGCCATGAACGAATTAGTAGTG and Sec13Halo-R CTGCAGATATTGTTCATATGTGTATT and cloned into the pHBIcHalo (46) expression vector using PstI and AsisI to generate pHBIPfSec13cHalo.

The plasmid pVSUN1-myc was generated in two steps. The plasmid pVB-myc (73) was used as a template. First, the selection marker was changed to BSD using the primers BSD-F (5′-GCGGCCGCAAAATGCCTTTGTCTCAAG-3′) and Hrp23′-R (5′-AACCAACGCGTTGGTGCAGTTTAAT-3′), and the sequence was amplified from pVBh (74) and inserted between the NotI and BstXI sites to generate the pV-myc construct. As a second step, PfSUN1 genomic sequence was amplified using SUN1HpaI-F (5′-AGTTAACATGAACATAAGCAACAG-3′) and SUN1KasI-R (5′-TTGGCGCCTTTAAATTTTCTTATAC-3′). The SUN1 1–190 fragment was amplified using SUN1HpaI-F and SUN1-190KasI-R (5′-GGCGCCTATTATTATTATTATTATATAGTGTAATAAATCCTG-3′). The SUN1Δ250–600 sequence was ordered as a gene block in pUC-57 (IDT) and cloned into the pV-myc construct with HpaI and KasI. PCR amplifications were performed using high-fidelity PrimeSTAR HS DNA polymerase (Takara Bio) or Q5 high-fidelity polymerase (New England Biolabs; NEB) utilizing NF54 genomic DNA as template, unless otherwise specified.

### Generation of transgenic lines and inducible knockdown

The PfSUN1-HA-glms and PfSUN2-HA-glms lines were created using the plasmid pSLI-HAx3glmS (75) as backbone. pSLI-HAx3glms was generated on the basis of the pSLI-2 X FKBP-GFP construct (27). The homology region for PfSUN1 (PF3D7_1215100) was cloned using the primers SUN1HR-Long-F (5′-GCGGCCGCTAATGGGCACTTGAATCT-3′) and SUN1-3UTR-R (5′-CTATATATTTTTGTTGGTACCACATG-3′) (NotI and XmaI). The homology region of PfSUN2 (PF3D7_1439300) was cloned using the primers SUN2HR-Long-F (5′-GCGGCCGCTAAACGTTAGTTGATAAAATAAAAACTATCG-3′) and SUN2-3UTR-R2 (5′-TAATAACTGTTGTGAAAAGTTTCTAACAGC-3′; NotI and XmaI).

Analysis of the integrated construct was performed using diagnostic PCR at the integration sites followed by sequencing and Southern blots of selected subclones.

For inducible knockdown, PfSUN1-HA-glmS and PfSUN2-HA-glmS parasites were grown in the presence or absence of 5 mM D-(+)-Glucosamine hydrochloride (GlcN; Sigma) over a 72 h time course. Following treatment, parasites were released by saponin lysis and sequentially analyzed in western blot.

### Western blotting

To collect parasite proteins, infected RBCs were lysed with saponin, and parasites were pelleted down by centrifugation. The parasite pellet was subsequently washed twice with PBS and lysed in 2 × Laemmli sample buffer or urea/SDS lysis buffer. The protein lysates were centrifuged, and the supernatants were subjected to SDS-PAGE (gradient, 4%–20%; Bio-Rad) and electroblotted onto a nitrocellulose membrane. Immunodetection was carried out by using mouse anti-GFP (1:1,000, Roche), mouse anti-HA primary antibody (1:1,000, Roche), rabbit anti-γH2A.X antibody (1:1,000, Cell signaling), and rabbit anti-aldolase (1:3,000, Abcam). The secondary antibodies used were goat anti-rabbit (1:10,000, Jackson ImmunoResearch) antibodies conjugated to horseradish peroxidase. The immunoblots were developed in EZ/ECL solution (Israel Biological Industries).

### X-ray irradiation of parasites and DNA repair assay

DNA damage of parasites and repair assay were performed as previously described (31). Briefly, tightly synchronized ring stage parasites were exposed to 6,000 rads X-ray irradiation using a PXi precision X-ray irradiator set at 225 kV and 13.28 mA. Immediately following irradiation, parasites were put back into culture to allow them to repair the damaged DNA. Protein was extracted 15 min after irradiation (0 h), as well as from parasites collected at 4 h after irradiation. Proteins extracted from untreated iRBCs were used as control. Western blot analysis was used to follow the changes in γ-*Pf*H2A compared with the housekeeping control gene aldolase in each treatment. These western blots were subjected to densitometry analysis to calculate the ratio between γ-*Pf*H2A levels and aldolase.

### Southern blot

Southern blots were performed as described in reference 24. Briefly, genomic DNA isolated from recombinant parasites was digested to completion by the restriction enzymes XbaI and BglII (PfSUN1-HA-glms) and XbaI and SacI (PfSUN2-HA-glms; NEB) and subjected to gel electrophoresis using 1% Agarose in Tris/Borate/EDTA. The DNA was transferred to a high-bond nitrocellulose membrane by capillary action after alkaline denaturation. DNA detection was performed using the DIG High Prime DNA Labeling and Detection starter kit (Roche). The HR sequences were amplified from pSLI-SUN1-HAx3-glms and pSLI-SUN2-HAx3-glms, DIG labeled, and used as probes.

## SUPPLEMENTAL MATERIAL

Supplemental Figures mbio.00216-25-s0001.pdf Figures S1 to S10.

ASM does not own the copyrights to Supplemental Material that may be linked to, or accessed through, an article. The authors have granted ASM a non-exclusive, world-wide license to publish the Supplemental Material files. Please contact the corresponding author directly for reuse.
